# Prevalence and Challenges of Hypertensive Heart Diseases in the Real World

**DOI:** 10.1155/2019/5430358

**Published:** 2019-06-10

**Authors:** Kai Hu, Chengxing Shen, Qin Yu

**Affiliations:** ^1^Department of Internal Medicine I, University Hospital Würzburg, Würzburg, Germany; ^2^Comprehensive Heart Failure Center (CHFC), University of Würzburg, Würzburg, Germany; ^3^Department of Cardiology, Shanghai Jiao Tong University Affiliated Sixth People's Hospital, 600 Yishan Road, Shanghai 200233, China; ^4^Affiliated Zhongshan Hospital of Dalian University, Dalian, Liaoning, China

Hypertension is the leading cause of global mortality and morbidity and remains the major and relatively easy preventable disease. However, investigation of prevalence and targeted efficient intervention of hypertensive heart diseases have been neglected in varying degrees over the past few years.

In this context, the studies of prevalence of hypertensive heart diseases and the potential of novel treatment and their challenges to combat and treat hypertensive heart diseases in the real world have attracted the interest of many scientists.

Liu et al. compared the prevalence of hypertension between the island and rural residents in Dalian, China. They performed modified MONICA questionnaire survey and found that prevalence of hypertension is extremely high in surveyed residents in island and rural areas of Dalian city. Moreover, awareness, treatment, and control rate of hypertension is much lower in surveyed residents than national level.

Coincidently, Khader et al. used a multistage sampling technique to select a nationally representative sample of adults from the population of Jordan. They showed that almost one third of Jordanian adults suffer from hypertension. Dismayingly, there was nonsignificant decrease in hypertension prevalence over nearly one decade. However, it is important to note that the rate of hypertension awareness increased significantly among men and women.

Whilst Aya and Hussain investigated the trend in the prevalence of hypertension in Greater Beirut Area, they found it to be consistent and relatively high, yet there was an observed improvement in the awareness and control of the disease.

It is well documented that hyperactivity of the sympathetic nervous system contributes a pivotal role in the pathophysiology of hypertension. Heart rate variability (HRV) and heart rate turbulence (HRT) reflect the autonomic regulation of cardiac function. Yu et al. explored the relationship between blood pressure control and autonomic nervous function assessing by HRV and HRT in hypertensive patients and demonstrated that impaired autonomic nervous function in hypertensive patients.

Recent clinical studies have shown that there are some controversies on the efficacy of RDN (renal denervation) in the treatment of hypertension. In the study by Li et al., data clearly demonstrated that the new RDN system is safe and could effectively reduce blood pressure in hypertensive patients in the absence of antihypertensive medications.

When it comes to screening LVH (left ventricular hypertrophy) in population, echocardiography is the current “gold standard” yet not an appropriate method for public screening. Hence, Park and Chon explored the effectiveness of combination of cardiothoracic ratio (CTR) in chest X-ray and well-known risk factors besides electrocardiography in asymptomatic hypertensive individuals. The authors showed that summing up the number of the risk factors of female, age≥ 65 y, BMI≥ 25 kg/m^2^, SLVA≥ 35 mm, and CTR≥ 0.50 may be a better diagnostic tool for screening LVH than the electrocardiography-only criteria, at the score≥ 2.

Besides essential hypertension, whether there are similar hemodynamic abnormalities that antedate the onset of fixed hypertension remains obscure. In the study by Ting et al., the answer is yes! This supports the notion that the elevation of blood pressure in hypertension may represent a later manifestation of an already abnormal vascular system rather than the vascular abnormalities being a result of the hypertension.

Risk factor profiles, clinical manifestations, and prognosis might differ between young patients with acute coronary syndrome (ACS) and elderly ACS patients. Ge et al. performed a retrospective and nonrandomized single center study and suggested that hypertension serves as an independent risk factor of multiple vessel disease and related to higher MACE rate during the short-term follow-up in young adults with ACS.

Although it is obvious from the studies included in this Special Issue that many advances have been made in clinical research highlighting the importance of improving the awareness of prevalence and taking effective and targeted intervention to combat the hypertensive heart disease in the real world, there are many grave issues still on the way, which need to be overcome. Firstly, the epidemiologic studies were performed in China, Jordan, and Lebanon, which were located in Asia, which cannot represent global status. Efforts should be launched to address the epidemiological surveys around the whole world. Secondly, although the awareness and control of hypertension have been promoted in the real world, the prevalence of this disease has not experienced a significant decrease or is even extremely high in surveyed area. Therefore, it is an urgent task for us to mount a comprehensive attack on hypertensive heart disease, harnessing all available resources to slow, arrest, and possibly even reverse the epidemic of hypertension.

Overall, the path ahead to mitigate the burden of hypertensive heart disease in real world is long and daunting, but watchful waiting is not an option. The progress reported in this Special Issue provides a relatively comprehensive prospect of hypertensive heart disease over the world highlighting the urgent task for us to take feasible strategies to effectively fight the hypertension and reduce the disease burden around the world.

